# Occurrence and Biosynthesis of Alkyl Hydroxycinnamates in Plant Lipid Barriers

**DOI:** 10.3390/plants6030025

**Published:** 2017-06-30

**Authors:** Frédéric Domergue, Dylan K. Kosma

**Affiliations:** 1 Laboratoire de Biogenèse Membranaire, UMR5200 CNRS/Université de Bordeaux, 33140 Villenave d’Ornon, France; frederic.domergue@u-bordeaux.fr; 2 Department of Biochemistry and Molecular Biology, University of Nevada, Reno, NV 89557, USA

**Keywords:** alkyl hydroxycinnamate, suberin, plant cuticle, HXXXD-motif/BAHD acyltransferase, fatty acyl reductase

## Abstract

The plant lipid barriers cuticle and suberin represent one of the largest biological interfaces on the planet. They are comprised of an insoluble polymeric domain with associated organic solvent-soluble waxes. Suberin-associated and plant cuticular waxes contain mixtures of aliphatic components that may include alkyl hydroxycinnamates (AHCs). The canonical alkyl hydroxycinnamates are comprised of phenylpropanoids, typically coumaric, ferulic, or caffeic acids, esterified with long chain to very long chain fatty alcohols. However, many related structures are also present in the plant kingdom. Although their functions remain elusive, much progress has been made on understanding the distribution, biosynthesis, and deposition of AHCs. Herein a summary of the current state of knowledge on plant AHCs is provided.

## 1. Introduction

**Figure 1:**
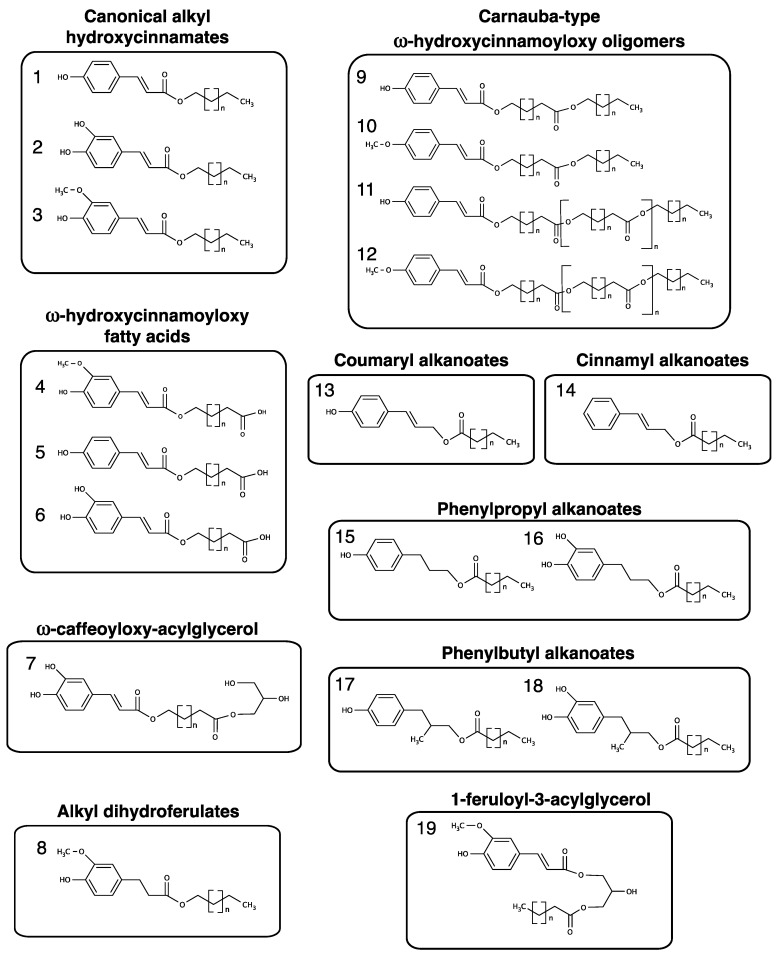
General structures of known alkyl hydroxycinnamates and related compounds. Chain lengths (*n*) vary depending on species, organ, and tissue. Numbers 1–19 are used to identify these structures as they are described in the text.

Alkyl hydroxycinnamates (AHCs), typically comprised of the phenylpropanoids coumaric, ferulic, or caffeic acids esterified with fatty alcohols (Figure 1, structures 1–3), have been described in many plant species. They are often found in association with plant cuticles and suberin. Cuticles are comprised of cuticular waxes and a lipid polymer termed cutin. Homologous series of very long chain alkanes, primary alcohols, fatty acids, as well as mid-chain ketones and secondary alcohols are considered typical cuticular waxes. Both suberin and cutin polymers are comprised of esterified long chain to very long chain mono, bi, and poly-functional lipids and glycerol. Long chain and very long chain waxes are also found associated with suberin. When associated with suberized tissues they are often referred to as components of suberin-associated waxes [1,2]. Although most recent studies have focused on their association with suberized layers, AHCs are also commonly found in many other types of tissues. For example, AHCs have been described in plant cuticles and leaf fibers. AHCs can also be comprised of phenylpropanoids esterified with the ω-hydroxyl group of ω-hydroxy fatty acids, i.e., ω-hydroxycinnamoyloxy fatty acids (Figure 1, structures 4–6). In addition, a number of less common AHC-related compounds have been identified including phenylpropanoids esterified to other hydroxyl-containing compounds like glycerol or hydroxycinnamyl alkanoates, among others. Although they have been described for decades, AHCs have received more attention in recent years with the discovery and characterization of the first genes encoding enzymes that function in their biosynthesis.

## 2. The Occurrence of Alkyl Hydroxycinnamates

There are many reports of AHC esters as natural products in diverse tissue types and in many different plant species. AHCs have been reported in more than 50 plant species and are likely even more widespread in the plant kingdom. Given that AHCs have been found in extant cycads like *Cycas vespertilio* [3] and many other gymnosperms [2], it can be hypothesized that their biosynthesis originated with, if not before, the appearance of gymnosperms. However, since fatty alcohols and hydroxycinnamates have been found in the cuticle of extant plants, whose appearance in evolutionary time predates that of gymnosperms (e.g., *Physcomitrella patens*) [4], it is likely that the capacity for plants to synthesize AHCs evolved more than 300 mya.

### 2.1. AHCs in Plant Cuticles, Suberin, and Other Tissues or Cell Types

AHCs in the form of alkyl coumarates, ferulates, and/or caffeates (structures 1–3 in Figure 1) have been reported most often, but many related structures have also been described in certain plants. Albeit recent works have discussed AHCs from a root suberin-centric viewpoint, AHCs have also been identified as components of bark, cuticular waxes, and in specialized cells like leaf fibers and trichomes.

#### 2.1.1. AHCs in Plant Cuticles

Although less common, a few reports have identified AHCs as components of cuticular waxes. For example, Santos et al. [5] identified C_16_–C_32_ alkyl coumarates as comprising ~6 wt. % of the cuticular wax extracted from English Ivy (*Hedera helix*) leaves (Figure 1, structure 1). Similarly, 13% of leaf waxes of the common beech tree (*Fagus sylvatica*) were comprised of C_18_–C_28_ alkyl coumarates [6]. Recently, both alkyl coumarates and alkyl ferulates (Figure 1, structure 3) were identified in total lipid extracts from the leaves, roots, and detritus (dead plant matter) of two species of cattails, *Typha domingensis* and *Typha latifolia* [7]. Whereas AHCs found in leaves could reside in any cell type, the evidence from other plant species supports a hypothesis that the AHCs found in *Typha* spp. leaves are derived from the cuticle. Curiously, *T. latifolia* leaves contained roughly 8-fold more AHCs than *T. domingensis* (~101 and 12 μg/g dry weight, respectively). *T. latifolia* leaves were dominated by alkyl coumarates whereas *T. domingensis* leaf AHCs were dominated by alkyl ferulates. AHCs with odd carbon chain length alkyl groups were identified in the leaves of both *T. latifolia* and *T. domingensis*. Although somewhat rare, AHCs with odd chain length alkyl groups have been found in the suberized layers of several plant species including rutabaga (*Brassica napus* subsp. rapifera), tobacco (*Nicotiana tabacum*), rapeseed (*Brassica napus* var. napus), the model plant Arabidopsis (*Arabidopsis thaliana*), salt cress (*Eutrema salsugineum*), and the evergreen tree *Tamarix aphylla*, among others [8,9].

#### 2.1.2. AHCs in Bark and Other Periderms

AHCs are commonly found in the stem periderm (bark) of a large number of woody species. Perhaps this is not surprising as tree bark can contain up to 50 wt. % suberin depending on the plant species [10,11]. In a review by Kolattukudy and Espelie [2], a large number of gymnosperm species were shown to contain alkyl ferulates in their bark wax extracts. Most of the alkyl ferulate-containing gymnosperm species comprised members of the Pinaceae, however, one species from the Podocarpaceae was also described. Douglas fir (*Pseudotsuga menziessii*) is one example of a Pinaceous species with an alkyl ferulate-enriched bark. *P. menziessii* was shown to contain C_22_ and C_24_ alkyl ferulates that represented 25% of the total wax extract equating to 1.4% of total bark dry weight. In general, the species described by Kolattukudy and Espelie contained C_16_–C_28_ ferulates with C_22_ and C_24_ alkyl chain lengths predominating.

AHCs are not only found in the bark of gymnosperms. Bark wax extracts from angiosperm species of the plant families Fabaceae, Myrtaceae, Podocarpaceae, and Salicaceae, among others, are known to contain alkyl ferulates [2]. For example, the bark of the fabaceous species *Erythrina stricta* was shown to contain C_25_, C_26_, and C_32_ ferulates, with C_32_ ferulates comprising the largest proportion of the alkyl ferulates [12]. Similarly, *Erythrina suberosa* was shown to contain C_14_–C_20_ alkyl ferulates [13]. In the case of *E. suberosa*, C_18_ alkyl groups dominated the alkyl ferulates. Alkyl ferulates have also been identified in the bark of myrtaceous *Eucalyptus* spp. [14,15,16]. The bark from four hybrids of *Eucalyptus*, grown in Brazil for the pulping/paper industry, was shown to contain C_22_–C_28_ alkyl ferulates, with alkyl ferulate totals ranging from 9 to 52 mg/kg of wood [14]. These alkyl ferulates likely originate from the bark and, as such, the mg/kg values would be much higher if normalized to kg of bark instead of kg of wood. In a separate study, C_22_, C_26_, and C_28_ ferulates as well as C_22_ and C_26_ ω-feruloyloxy aliphatics (Figure 1, structure 4) were present in dichloromethane (CH_2_Cl_2_) extracts of *Eucalyptus globulus* bark [15]. A later study by Freire et al. [16] demonstrated the presence of C_24_ ferulate as well as C_22_–C_28_ coumarates in *E. globulus* bark.

Although many woody species contain alkyl ferulate bark waxes, there are species whose periderm wax is dominated by other classes of AHCs. For example, the bark of three species of acacia (Fabaceae family), *Acacia dealbata*, *A. melanoxylon*, and *A. retinodes*, were shown to contain predominantly alkyl caffeates (Figure 1, structure 2) with minor amounts of alkyl coumarates and ferulates.

Potato (*Solanum tuberosum*) is perhaps the species in which AHCs have been best described [17,18,19,20]. Native and wound periderm of potato tubers, which are modified stems, both contain alkyl ferulates. Wound periderm is described as containing C_16_–C_28_ alkyl ferulates by Bernards and Lewis [17] and C_16_–C_32_ by Schreiber et al. [19]. Native periderm is also described as containing C_16_–C_32_ alkyl ferulate waxes including odd chain homologs [19,20,21,22,23]. In addition, C_16_–C_30_ alkyl ferulates have been described in potato “pulp” or starchy parenchyma cells [18]. Whether this was a result of periderm contamination of the pulp remains to be determined. Discrepancies in chain lengths between different reports likely represent cultivar to cultivar differences and differences in environmental conditions.

#### 2.1.3. AHCs in Suberized Root Tissues

The endodermis of young roots as well as the periderm of mature roots are suberized tissues. The model plant Arabidopsis has proven to be very useful for understanding the molecular genetics and biochemistry of AHC synthesis. Li et al. [24] made what likely represents the first identification of AHCs in Arabidopsis. Alkyl caffeates, coumarates, and ferulates were identified in extracts of mature roots submerged in chloroform (CHCl_3_) for a short period of time. Molina et al. [25] provided a second description of AHCs in mature Arabidopsis roots. Since Li’s and Molina’s studies, Kosma et al. [26] and Vishwanath et al. [27] have identified three genes encoding the enzymes responsible for the synthesis of the fatty alcohol comprising the alkyl group as well as the acyltransferase responsible for the synthesis of alkyl caffeates (discussed further in Section 3). More recently, it was shown that suberin and associated waxes represent up to 55 mol. % of all fatty acyl chains in Arabidopsis roots [28]. From these works, it can be estimated that the alkyl groups of AHCs comprise 9–13% of all fatty acyl chains in Arabidopsis roots depending on the age of the plant. Considering that nearly all cells contain lipidic endomembrane systems and that suberin is essentially restricted to two cell types in the roots, endodermis and periderm, it can be said that AHCs themselves represent a major sink for fatty acyl metabolism in Arabidopsis roots. A close relative of Arabidopsis, Camelina (*Camelina sativa*), was found to have a root wax composition similar to that of Arabidopsis [29]. Typical AHC classes were found in Camelina root wax extracts (alkyl caffeates, coumarates, and ferulates) with alkyl caffeates dominating. C_18_ and C_20_ were the dominant alkyl groups in Camelina root AHCs.

The root periderm waxes of two solanaceous species have also been characterized [8,30]. Both tobacco (*Nicotiana tabacum*) and tomato (*Solanum lycopersicum*) were shown to contain only alkyl ferulates in their root wax AHCs. Potato periderm waxes, also a member of the Solanaceae, contain alkyl ferulates as the sole AHC class even though the potato tuber is technically a modified stem. Curiously, periderm wax extracts from the tuberous roots of sweet potato (*Ipomoea batatas*), a member of the same order as potato, tobacco, and tomato (Solanales), contained all three classes of AHCs (alkyl caffeates, coumarates, and ferulates) but was dominated by alkyl coumarates. Kosma et al. [8] also found that root wax extracts from the monocotyledonous plants rice (*Oryza sativa* subsp. japonica) and maize (*Zea mays*) contained only alkyl ferulate AHCs. Notably, the chain length distribution differed quite substantially; maize contained C_18_–C_22_ ferulates, with C_22_ ferulates dominating, whereas rice contained C_20_–C_28_ ferulates, with C_26_ ferulates dominating.

One question that comes to mind with regard to AHCs is whether any chemotaxonomic relationships exist between composition and species. Kosma et al. conducted a survey of root periderm/exodermis wax composition from diverse plant species [8]. In this study, it was shown that AHCs are common among distantly-related taxa albeit with differing compositions. Little could be inferred on the relationship between AHC composition and taxonomy with the exception of a few general observations. Brassicaceae AHCs were dominated by either alkyl coumarates or alkyl caffeates and contained little if any alkyl ferulates. Alkyl chain lengths ranged from C_18_–C_22_. *Raphanus* spp. like daikon (*Raphanus sativus* var. niger or var. longipinnatus) and radish (*Raphanus sativus* var. sativus) contained both alkyl coumarates and alkyl caffeates, with alkyl coumarates predominating; C_18_ was the dominant alkyl chain length. Alkyl caffeates comprised the majority of AHCs in rapeseed (*Brassica napus* var. napus), Arabidopsis, Rutabaga (*Brassica napus* supbs. rapifera), and salt cress (*Eutrema salsugineum*), with C_22_ alkyl chain lengths predominating. Peas (*Pisum sativum*), on the other hand, contained exclusively alkyl coumarates. Although it is tempting to infer chemotaxonomic relationships based on these and other observations, one must exercise caution. It is quite probable that AHC composition and content is influenced by environmental factors. As a case in point, it was recently shown that age has a marked impact on the makeup of AHCs in the roots of Arabidopsis [28]. Similarly, it has been shown that NaCl-treatment elevates the amounts of a specific class of AHCs, alkyl coumarates, in Arabidopsis taproots [26].

#### 2.1.4. AHCs in Other Tissues or Cell Types

Even though AHCs have been described primarily in cuticle and suberized cell layers, they have also been identified in other tissues in a number of natural products-oriented publications. In these studies, AHC-containing extracts were derived from whole plants or whole organs, making it difficult to determine their cell or tissue-specific origin. For example, C_18_–C_28_ alkyl coumarates have been identified in CHCl_3_ extracts of whole *Artemisia campestris* plants [31]. However, there are some descriptions of AHCs in specific tissues or organs other than cutinized or suberized tissues. There are at least two examples of AHCs described in leaf fibers. Alkyl coumarates (C_20_–C_28_), ω-coumaroyloxy fatty acids (C_22_–C_28_; Figure 1, structure 5), alkyl ferulates (C_20_–C_28_), and ω-feruloyloxy fatty acids (C_22_–C_28_) have been identified from the leaf fibers of abaca (*Musa textilis*) [32]. Similarly, acetone extracts of sisál (*Agave sisalana*) fibers were shown to contain 9.15 mg of C_18_–C_30_ alkyl ferulates and 0.25 mg of C_22_–C_30_ ω-feruloyloxy fatty acids per 100 g of dry leaf fiber [33].

Whereas Kosma et al. [8] identified AHCs in sweet potato periderm extracts, alkyl ferulates have been detected in other parts of sweet potato plants. For example, Kawanishi et al. [34] determined that C_16_–C_18_ alkyl ferulates are present in both the flesh and periderm of three different cultivars of sweet potato. Additionally, Snook [35] demonstrated that the root and vine latex of sweet potato contained C_16_–C_20_ alkyl coumarates. In this study, it was shown that 1.5–3% of vine and 10.6% of the root latex mass, respectively, was comprised of alkyl coumarates; C_18_ alkyl coumarates dominated vine latex whereas roughly equal proportions of C_16_ and C_18_ alkyl coumarates were found in root latex. Snook also detected trace amounts of C_16_ ferulates in sweet potato latex. These findings are similar to the findings of Kosma et al. [8] in which alkyl coumarates comprised ~7% of sweet potato periderm wax AHCs.

Cotton (*Gossypium hirsutum*) fibers present an interesting case. Botanically speaking, cotton fibers are considered a form of modified epidermal cell or trichome. Whereas white cotton fibers are mainly comprised of cellulose overlaid with a cuticle [36,37,38], green cotton fibers are known to be heavily suberized, presenting lamellae, monomers, and waxes typical of suberin [39,40,41,42]. Consistent with a suberin-associated deposition of AHCs, Schmutz et al. [39] isolated and identified a novel AHC trimer from green cotton fibers that contained a ω-caffeoyloxy fatty acid (Figure 1, structure 6). This trimer was identified to be ω-caffeoyloxy-docasanoylglycerol (Figure 1, structure 7). Even though this trimer is found in organic solvent extracts and therefore would usually be classified as a wax, this ω-caffeoyloxy acylglycerol may represent an intermediate of suberin polymer assembly, as its structure is reminiscent of the structures released from partial depolymerization of suberized periderms [43].

### 2.2. Atypical AHCs and AHC-Related Compounds

A variety of atypical AHCs and AHC-related compounds have been identified in soils and several plants species. For example, alkyl esters of dihydroferulate (Figure 1, structure 8) have been found in soil extracts. Alkyl dihydroferulates represent a novel class of AHCs, containing phenylpropanoates instead of phenylpropenoates [7]. Whether reduction (hydrogenation) of the double bond at the α carbon of the propenoate, ferulate, occurs in planta or ex planta remains unknown. Another example of an atypical AHC is found in the leaf waxes of the carnauba palm (*Copernica prunifera*). Carnauba leaf waxes are commonly used as furniture polish and in other industrial applications. Carnauba waxes of the T1 type are reported to contain 21 wt. % of trimeric diesters of coumaric acid and 7 wt. % of trimeric diesters of 4-methoxycinnamic acid (Figure 1, structures 9 and 10) [44,45,46]. These esters are comprised of coumarate or 4-methoxycinnamate esterified to the ω-hydroxyl group of an ω-hydroxy fatty acid. The carboxylate end of the ω-hydroxy fatty acid is then esterified to a fatty alcohol. 4-Methoxycinnamate fatty alkyl esters have rarely been reported, representing another novel class of AHCs. T3 type carnauba wax is reported to contain coumarate and 4-methoxycinnamate esters with higher degrees of polymerization than those observed in T1 type wax (Figure 1, structures 11 and 12). These and the other oligomeric waxes of carnauba are reminiscent of what one could expect for intermediates of cutin polymer assembly.

A number of alkyl hydroxycinnamate-related wax compounds have also been reported. One example is found in reports on apple (*Malus domestica*) fruit cuticular waxes. Apple fruits were found to contain esters of the monolignol coumaryl alcohol esterified with saturate C_16_–C_26_ fatty acids (coumaryl alkanoates; Figure 1, structure 13) [47]. In a separate study, coumaryl alkanoates with chain lengths from C_18_–C_24_ were identified in apple fruit cuticular waxes [48]. Notably, in this study, coumaryl alkanoate esters of oleate, linoleate, and linolenate (C_18:1_, C_18:2_, and C_18:3_) were identified. Similar compounds have been described in wax extracts from the flowers of faba bean (*Vicia faba*) [49]. Cinnamyl alcohol and cinnamyl alkanoates (Figure 1, structure 14) with alkanoate chain lengths ranging from C_16_–C_24_ were found to be the dominant wax constituents of faba bean flowers. Yew (*Taxus baccata*) needle waxes contain several interesting AHC-related compounds [50]. Unlike the coumaryl alkanoates found in apple fruit waxes, yew wax extracts contained phenlypropyls and phenylbutyl alkanoates instead of phenylpropenyl alkanoates. Fatty acid chain lengths ranged from C_18_–C_28_. The phenylpropyl groups comprised 3-(4’-hydroxyphenyl)-propanyl and 3-(3’,4’-dihydroxyphenyl)-propyl groups (Figure 1, structures 15 and 16, respectively); phenylbutyl groups comprised 4-(4’-hydroxyphenyl)-2-butyl and 4-(3’,4’-dihydroxyphenyl)-2-butyl groups (Figure 1, structures 17 and 18, respectively).

One unusual alkyl ferulate found in the bark of *Tamarix aphylla* consists of a ferulate esterified to the *sn*-*1* hydroxyl of a molecule of glycerol with a C_25_ fatty acid esterified to the *sn*-*3* hydroxyl of the same glycerol molecule (Figure 1, structure 19) [9]. This brings to light an important question about whether hydroxyl group-containing molecules, other than fatty alcohols, can serve as acyl acceptors for the HXXXD-motif/BAHD acyltransferases that catalyze AHC formation. Glycerol is well known to be a component of suberin and glyceryl ferulates have been detected in the partial depolymerization products of suberin [51,52], thus, it is plausible that glycerol could be esterified with hydroxycinnamates via the activity of HXXXD-motif/BAHD acyltransferases [40,52,53]. As a case in point, Kim et al. [54] identified a rice HXXXD-motif/BAHD that was most highly expressed in roots and exhibited hydroxycinnamoyltransferase activity with glycerol as an acyl acceptor in vitro. Notably, this enzyme could use feruloyl, coumaroyl, and caffeoyl CoA acyl donors and add hydroxycinnamoyl groups to both the *sn*-*1* and *sn*-*2* position of glycerol.

## 3. The Biosynthesis of Alkyl Hydroxycinnamates

Given that most AHCs are comprised of a phenylpropanoid (coumaric, ferulic, or caffeic acid) esterified with fatty acid derivatives (a fatty alcohol or an ω-hydroxy fatty acid), their biosynthesis can be divided in three major steps. The phenylpropanoid pathway provides the hydroxycinnamates, reduction of a fatty acyl chain yields the alkyl, and finally HXXXD-motif/BAHD-type acyltransferases combine both moieties to produce AHCs. All these steps occur in the cytosol and/or the endoplasmic reticulum.

### 3.1. Biosynthesis of Hydroxycinnamates

Coumaric, ferulic, and caffeic acids are produced by the phenylpropanoid pathway, which is also the origin of plant cell wall components like lignin, and therefore has been the focus of numerous studies for decades. Since many detailed reviews on the phenylpropanoid pathway and the biosynthesis of lignin are available [55,56], this review only briefly describes the biosynthesis of the hydroxycinnamates found in AHCs.

The synthesis of hydroxycinnamates starts with phenylalanine, a product of the shikimate pathway. Phenylalanine is converted to cinnamic acid by phenylalanine ammonia-lyase (PAL). Hydroxylation at C4 on the phenyl ring, via the activity of cinnamate 4-hydroxylase (C4H), gives rise to *p-*Coumaric acid. *p-*Coumaroyl-CoA and *p-*Coumaroyl shikimate/quinate serve as intermediates for the subsequent hydroxylation by *p-*Coumarate 3-hydroxylase (C3H) that leads to the formation of caffeoyl-CoA. The enzymes 4-Coumarate:CoA ligase (4CL) and *p*-hydroxycinnamoyl-CoA:quinate/shikimate *p*-hydroxycinnamoyltransferase (HCT) form these intermediates. Finally, methylation of the hydroxyl group at position 3 of the phenyl ring by the caffeoyl-CoA *O*-methyl-transferase (CCoAOMT) results in the production of feruloyl-CoA.

### 3.2. Reduction of Fatty Acyl-Chains

**Figure 2:**
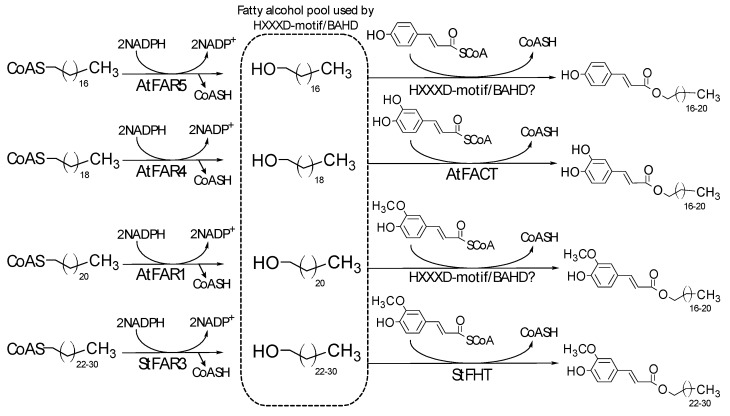
General scheme of the alkyl hydroxycinnamate (AHC) biosynthesis pathway. Whereas Fatty Acyl Reductases (FARs) have clear acyl-chain length preferences (reactions on the left), HXXXD-motif/BAHD acyltransferases utilize a range of alkyl-chain length as substrates (reactions on the right) with some degree of specificity for hydroxycinnamoyl CoAs.

The alkyl chains of AHCs are usually long- (C_16_, C_18_) to very long- (≥C_20_) chain saturate fatty alcohols, (Figure 1 and Figure 2), which are derived from corresponding fatty acid homologs. C_16_ and C_18_ fatty acids are produced de novo in the plastid by the Fatty Acid Synthase (FAS) complex, whereas ≥C_20_ fatty acids are formed through elongation of C_16_ and C_18_-CoAs in the endoplasmic reticulum (ER) by the fatty elongase complex (FAE) [57]. β–Ketoacyl CoA Synthase 2 (KCS2) and 20 (KCS20) components of the ER FAE complex produce the C_22_ and C_24_ acyl chains found in suberin [58,59]. Once elongated to specific chain lengths, fatty acyl-CoAs are reduced to the corresponding fatty alcohols by Fatty Acyl Reductases (FARs) in the presence of nicotinamide adenine dinucleotide phosphate (NADPH) as a reducing equivalent [60]. All FAR proteins display a Rossmann-fold NAD(P)H binding domain at their *N*-terminus and a fatty acyl-CoA reductase (FAR_C) domain at their *C*-terminal end. Most FARs are predicted to contain transmembrane domain(s) and are purported to have ER localization, although soluble plastidial FARs acting on acyl-ACPs have also been described [60].

In Arabidopsis, three FARs were shown to be involved in the production of the fatty alcohols present in the root suberin polymer and associated waxes [26,27,28,61]. Heterologous expression in yeast and analysis of single *far* mutants indicated that FAR5, FAR4, and FAR1 are primarily responsible for the production of C_18_, C_20_, and C_22_ fatty alcohols, respectively, although FAR4 and FAR1 do exhibit some overlapping activity with regard to acyl chain length [61]. In triple *far1far4far5* mutant lines, obtained through an amiRNA approach (FAR4 and FAR5 genes being positioned in tandem on chromosome 3), the amount of soluble fatty alcohols and AHCs present in root waxes was reduced by more than 80% [27,28].

Other plant species, like potato, contain large amounts of alkyl ferulates in their periderm with alkyl-chain length ranging from 16 to 32 carbons including some odd-numbered carbon alkyl chains [17,19]. To achieve the formation of the most abundant C_28_ to C_30_ fatty alcohols, additional FAR homologs must be present. The obvious candidate for a FAR that would form these very long chain fatty alcohols is an ortholog of Arabidopsis FAR3/CER4. Arabidopsis FAR3 is involved in the formation of ≥C_24_ fatty alcohols, albeit it has been characterized in the context of cuticular wax biosynthesis [62]. Interestingly, a preliminary report on StFAR3, the potato homolog of Arabidopsis FAR3/CER4, indicated that this gene was not only expressed in leaves but also in tuber periderm, and that RNAi silencing of StFAR3 reduced the levels of >C_24_ alkyl ferulate periderm waxes [63].

### 3.3. Alkyl Hydroxycinnamate Formation

The transfer of CoA-activated hydroxycinnamic acid derivatives onto hydroxylated aliphatics is catalyzed by a subset of HXXXD-motif/BAHD enzymes [64]. Using wound-healing potato tuber discs, Lotfy and coworkers [65] first reported an enzymatic activity capable of transferring ferulic acid from feruloyl-CoA to ω-hydroxypalmitic acid (hydroxycinnamoyl-CoA: ω-hydroxypalmitic acid *O*-hydroxycinnamoyltransferase, HHT, EC 2.3.1.-). This enzyme also combined primary alcohols with ferulic acid, thereby producing AHCs. A similar activity was then described with an enzyme purified from tobacco suspension cells [66]. This enzyme had the highest specificity for feruloyl-CoA and ω-hydroxypalmitic acid even if sinapoyl-CoA, coumaroyl-CoA as well as several primary alcohols could be used as substrates. Lotfy et al. [67] also determined that feruloyltransferase activity with ω-hydroxypalmitic acid was widespread, being detected in 17 plant species from 11 different families of angiosperms and gymnosperms including both monocots and dicots. From the same study, feruloyltransferase activity with a fatty alcohol acceptor was demonstrated in 15 plant species. This finding is congruent with the pervasive presence of AHCs in higher plants (Section 2).

The first HXXD-motif/BAHD for which deregulation affected suberin-associated AHCs was cloned from potato and named FHT for ω-hydroxyacid/fatty alcohol hydroxycinnamoyl transferase [20]. The tuber periderms of FHT RNAi lines presented a 75% decrease in both ω-hydroxyoleic and ferulic acids. However, the total amount of polymerized suberin was unaffected. Additionally, the composition of suberin-associated waxes was strongly modified, with a concomitant decrease in alkyl ferulates and increase in free fatty alcohols as well as a doubling of total wax amount. In vitro assays with recombinant FHT protein demonstrated feruloyl transferase activity with both ω-hydroxypalmitic acid and medium chain (C_12_ and C_14_) fatty alcohols. The exact specificity and function of FHT with regard to suberin polymer vs. alkyl ferulate synthesis is nevertheless difficult to discern. The presumed native substrates, based on RNAi line phenotypes, are ω-hydroxyoleic acid or C_18_–C_30_ fatty alcohols, but FHT activity was never assayed with these specific acyl acceptors. Thus, it is not clear if FHT is involved in both suberin polymer and associated wax synthesis or if it is specific to suberin polymer (which represents 96% of the periderm lipids) biosynthesis with an indirect effect on alkyl ferulate waxes. Further obscuring the matter is the results obtained from the investigation of the Arabidopsis ortholog of FHT, Aliphatic Suberin Feruloyl Transferase or hydroxycinnamoyl-CoA: ω-hydroxyacid *O*-hydroxycinnamoyltransferase (ASFT/HHT). The root suberin of *asft* T-DNA insertion mutants was nearly devoid of polymerized ferulate, but root wax AHCs (including alkyl ferulates) were completely unaffected [25]. However, recombinant ASFT/HHT can perfectly well utilize C_8_–C_16_ fatty alcohols as acyl acceptors [26,64]. Curiously, using recombinant ASFT/HHT protein, Kosma et al. [26], Molina and Kosma [64], and Gou et al. [68] did not observe any measurable feruloyltransferase activity with a C_18_ alcohol acyl acceptor. Visual inspection of different chain lengths of fatty alcohols in assay conditions typically used for HXXXD-motif/BAHD acyltransferases demonstrated a clear precipitation of C_14_–C_18_ fatty alcohols with corresponding decreases in enzyme activity [26,64]. Similarly, a poplar (*Populus trichocarpa*) ortholog of FHT and ASFT/HHT exhibited no activity with C_16_–C_22_ fatty alcohol acyl acceptors, albeit high activity was observed with ω-hydroxy fatty acids [69]. A paralog of Arabidopsis ASFT/HHT, Deficient in Cutin Ferulate (DCF), also had little to no enzyme activity with long chain fatty alcohols [70]. As such, the absence of in vitro hydroxycinnamoyltransferase activity with long-chain fatty alcohols may not indicate that they are not substrates for a given HXXXD-motif/BAHD enzyme; more likely, it represents a problem of substrate solubility. Notably, ASFT/HHT was able to feruloylate the ω-hydroxyl of free ω-hydroxypalmitic acid as well as the ω-hydroxyl of methyl ω-hydroxypentadecanoate [25]. Although little is known about how aliphatic substrates bind to the catalytic site of HXXXD-motif/BAHD enzymes, the use of both free and methyl-esterified fatty acids as well as fatty alcohols (which do not contain an oxygenated functional group on the α end of the alkyl chain) suggests that ASFT/HHT may not recognize or discriminate the α terminus of aliphatic substrates. The question remains as to what dictates the specificity of an enzyme like ASFT/HHT for ω-hydroxy fatty acids, in planta, even though it is perfectly capable of utilizing fatty alcohols as acyl acceptors in vitro. With the same reasoning, it remains unclear what allows FHT to utilize both ω-hydroxy fatty acid and fatty alcohol acyl acceptors in planta even though it shares 79% amino acid sequence similarity with ASFT/HHT.

In contrast to the suberin-associated waxes of potato periderm, which contain only alkyl ferulate esters as AHCs, those of Arabidopsis are dominated by alkyl caffeates which represent roughly 80 mol. % of total waxes [8,28]. The enzyme responsible for their biosynthesis has been characterized using a classic reverse genetic approach and is referred to as FACT for fatty alcohol: caffeoyl-CoA caffeoyl transferase [26]. Roots from *fact* mutant plants displayed a near complete lack of chloroform-soluble alkyl caffeates esters, whereas only very minor changes in polymerized aliphatics were observed. As such, FACT likely represents the first HXXXD-motif/BAHD identified to be specifically involved in AHC biosynthesis. In vitro assays with recombinant FACT protein demonstrated a clear preference for a caffeoyl-CoA acyl donor even though coumaroyl and feruoyl-CoAs could also be used as substrates. Such assays also showed that FACT activity was inversely correlated with the fatty alcohol chain length, again suggesting that fatty alcohol solubility may affect in vitro determination of the native alkyl chain-length specificity of suberin or lipid-associated HXXXD-motif/BAHD-type enzymes [26].

A closer look at AHC chain length distribution in various plants does, however, suggest that HXXXD-motif/BAHD-type enzymes do have alkyl-chain substrate specificities. For example, the dominant alkyl chain lengths found in alkyl coumarates of *Typha latifolia* leaves were C_16_ and C_18_, whereas alkyl ferulates were dominated by C_16_ and C_20_ alkyl chains [7]. Similarly, in the bark of three species of acacia, alkyl chain lengths ranged from C_16_–C_28_ in the alkyl caffeates, C_24_–C_28_ in the alkyl ferulates, and C_24_–C_26_ in the alkyl coumarates [71]. These observations clearly illustrate a differential distribution of AHC classes within a given species and a differing range of alkyl chain lengths among the different AHC classes. It remains unclear which biosynthetic mechanism(s) is responsible for these differences but several hypotheses can be put forth. The greater protein abundance of a specific HXXXD-motif/BAHD acyltransferase might result in the prevalence of one AHC class over another. Differences in the catalytic efficiencies of the BAHD/HXXXD acyltransferase specific to each class of AHCs could also explain these distributions. The tight association of a fatty acyl-CoA reductase with a HXXXD-motif/BAHD acyltransferase (e.g., caffeoyltransferase) versus others (e.g., feruloyltransferase) could also favor the accumulation of specific AHC classes and chain lengths. Whether differences in AHC composition are due to protein stability, narrow substrate specificities, catalytic efficiency of HXXXD-motif/BAHDs, or proximity of enzymes within a metabolon remains to be discovered.

The three most common classes of AHCs are alkyl caffeates, coumarates, and ferulates (Figure 1). All three classes are present in Arabidopsis roots. Several questions on the biosynthesis of AHCs remain to be answered. For example, acyltransferases for the formation of alkyl ferulates and caffeates, FHT and FACT, respectively, have been identified. However, the acyltransferase, HXXXD-motif/BAHD or otherwise, for the specific synthesis of alkyl coumarates (i.e., the coumaroyl transferase) is yet to be identified. Second, if ASFT is not responsible for the synthesis of the alkyl ferulates found in root wax extracts, what enzyme is responsible for alkyl ferulate waxes in Arabidopsis? In this context, it is notable that *fact* mutants also had reduced amounts of alkyl ferulate root waxes. Thus, there is a distinct possibility that FACT and ASFT or FACT and a yet to be identified acyltransferase may work redundantly in the synthesis of alkyl ferulates.

### 3.4. AHC Export and Regulation

As of today, the intracellular trafficking and export as well as the transcriptional regulation of AHCs is poorly understood. However, a few studies suggest that some players involved in the synthesis and regulation of the suberin polymer also impact AHC content.

In Arabidopsis, three ABCG transporters, ABCG2, 6, and 20, were shown to be required for suberin biosynthesis, as the root suberin of the corresponding triple mutant was severely impacted in both polymerized aliphatics and root wax AHCs [72]. However, qPCR analysis showed that the expression of *FAR1*, which produces most of the fatty alcohols found in AHCs, *FACT*, which produces the major class of AHCs, as well as *FAR4*, was strongly reduced in *abcg2 abcg6 abcg20*, suggesting that AHCs synthesis, rather than export, may be impacted in these lines [28]. Additionally, Landgraf et al. demonstrated that silencing of a potato ABCG transporter (StABCG1) orthologous to Arabidopsis AtABCG1 and AtABCG16 led to the decreased incorporation of suberin monomers into the suberin polymer with a concomitant accumulation of feruloyloxy fatty acids, feruloyloxy acylglycerols, hydroxyalkyl ferulates (ferulate esterified to one hydroxyl of an α,ω-diol), α,ω-dicarboxylic acids, and other suberin-like monomers [22]. Nevertheless, biochemical demonstration of the transport of lipid components by these proteins is still missing.

A number of transcription factors involved in the regulation of plant cuticle biosynthesis have been identified, but none were shown to have any effect on the accumulation of AHCs [73,74,75,76,77]. Similarly, several transcription factors have been shown to regulate suberin deposition in Arabidopsis seed coats, AtMYB107 and AtMYB9 [78,79]. However, it is unclear if AtMYB107 and AtMYB109 regulate AHC biosynthesis and deposition or if Arabidopsis seed coats even contain AHCs. Nevertheless, the fact that the *far1far4far5* triple mutant seeds have permeability defects [27] strongly suggests that this the case. Cuticle-related transcription factors like *SHN3* are also expressed in the endodermis and thus are candidates for the regulation of AHC synthesis [75]. Similarly, GhMYB25 has been shown to regulate cotton fiber formation and may serve to regulate suberin and associated wax deposition in green cotton fibers [80]. Curiously, downregulation of the tomato gene *Defective in Cuticular Ridges* (*SlDCR*) resulted in intense suberization of tomato fruits including the accumulation of alkyl ferulate waxes [78].

Recently, however, the first transcription factor identified to activate suberin deposition, AtMYB41, was shown to regulate both suberin polymer and soluble AHC biosynthesis [81]. Ectopic expression of *AtMYB41* in Arabidopsis and *Nicotiana benthamiana* resulted in the formation of typical suberin lamellae, the deposition of nearly all molecular species of suberin monomers in leaves, as well as the presence of suberin-associated waxes including AHCs. More recently, another transcription factor from apple, MdMYB93, was shown, also by transient expression in *N. benthamiana*, to induce the formation of suberin polymer and associated waxes [82]. Collectively, these data support the hypothesis that the same regulatory processes may control the biosynthesis of both the suberin polymer and suberin-associated waxes including AHCs.

## 4. Biological Activities and Putative Functions of Alkyl Hydroxycinnamates

The specific biological roles of alkyl hydroxycinnamates in planta are presently unknown. However, they are known to function as antioxidants. The specific antioxidant activities of hydroxycinnamates is determined by several factors including the type and number of functional groups present on the phenyl ring as well as the presence or absence of an esterified alkyl chain [83]. Caffeic acid and derivatives tend to be quite efficient at scavenging radicals due to the presence and proximity of the hydroxyl groups at C3’ and C4’ which effectively form catechol. Ferulic and coumaric acids, and their derivatives, are also effective antioxidants, but generally to a lesser degree than caffeates. Caffeates, ferulates, and coumarates operate as antioxidants through forming quinone and semiquinone intermediates. The presence of esterified alkyl groups tends to improve the antioxidant activity of caffeates, whereas alkyl coumarates and ferulates have lower antioxidant activities than their free acid counterparts. Considering this, it can be hypothesized that alkyl hydroxycinnamates serve to protect their host organisms against oxidative stress. The protective mechanism may entail prevention of lipid peroxidation. Several reports have shown that AHCs can prevent lipid oxidation albeit with different efficiencies depending on AHC class and alkyl chain length [18,84,85]. Jayaprakasam et al. [86] demonstrated that long chain to very long chain (C_16_–C_22_) alkyl caffeates and short chain ferulates (C_3_–C_5_) were very effective inhibitors of lipid peroxidation at a concentration of 5 μg/mL, with activities nearly equal to that of commercial antioxidants commonly used as food additives.

There are several lines of evidence that point to allelopathy as another function of AHCs. C_22_–C_30_ alkyl ferulates from root extracts of *Kalanchöe daigremontiana* were shown to negatively impact the growth of plantlets from the same species [87]. The mechanism of alkyl ferulate toxicity is unclear, however, free ferulic acid is a known allelopathic phytochemical [88,89]. Liebl et al. [88] demonstrated that ferulic acid undergoes a bacterium-catalyzed decarboxylation reaction, forming a more phytotoxic styrene derivative, 2-methoxy-4-ethenylphenol. Nair et al. [87] hypothesized that alkyl ferulates are cleaved to free ferulate which can then act in the soil as an allelochemical. Not only is there evidence of alkyl ferulates as phytotoxic chemicals, coumaric acid has also been implicated to function as an allelochemical. Rasumssen et al. [89] demonstrated that both ferulic and coumaric acids exhibited allelopathic effects on sorghum (*Sorghum bicolor*) seedlings in terms of both inhibition of seed germination and seedling growth. They further demonstrated that equimolar mixtures of ferulic and coumaric acids exhibited synergistic effects on seedling growth inhibition. Surprisingly, dilute concentrations of either ferulic acid or coumaric acids had stimulatory effects on plant growth, suggesting that hydroxycinnamates may act in a hormetic manner. The synergistic, allelopathic effect of ferulic and coumaric acid on plant growth could explain why some plant species possess multiple classes of AHCs.

AHCs may also function as antimicrobial compounds. In two separate studies, Baranowski and Nagel [90,91] demonstrated that methyl esters of caffeates, coumarates, and ferulate, as well as ethyl (C_2_) and propyl (C_3_) esters of caffeate, caused significant delay of *Pseudomonas fluorescens* growth in liquid cultures. They further demonstrated that this growth inhibition was likely due to an inhibition of cellular respiration and resulting depletion of cellular energy (ATP) and not just general membrane-disrupting effects observed with other antimicrobial phenolics. Little if any information on the antimicrobial activities of the long chain and very long chain AHCs can be found in the literature. However, it was recently shown that four mutant lines affected in key enzymes involved in the biosynthesis of suberin (far1 far4 far5*, *kcs2 kcs20*, *asft*, and *fact*) presented an increased susceptibility to *Ralstonia solanacearum* [92]. Although preliminary, these data showed that the mutants that were more specifically affected in AHCs (far1 far4 far5* and *fact*) had the lowest survival rates, further suggesting a role for AHCs in plant-microbe interactions, perhaps as defense compounds. More than a decade ago, annual crop loss due to plant pathogens in the USA reached approximately $33 billion [93]. Understanding the putative function of AHCs as preemptive compounds for plant defense against microbial invaders represents an important and unexplored line of research. Unravelling the exact function of AHCs in plant-microbe interactions may provide long-term strategies for reducing the growing problem of crop loss from microbial pathogens.

## 5. Conclusions

In the past 50 years, numerous studies on suberized barriers have reported the presence of canonical AHCs in a vast array of plant species. A careful examination of the literature reveals that canonical AHCs are not limited to suberized tissues, but can also be found in cuticular waxes that cover the epidermis of all plant aerial organs and in the leaf fibers and trichomes of distantly-related species. Similarly, it can be surmised that many AHC-related compounds apart from the canonical AHCs are found in numerous plant species and are also not restricted to suberized tissues. Given the nearly ubiquitous presence and rather widespread distribution of AHCs in the plant kingdom, as well as their evolutionary conservation for millions of years, it can be inferred that such compounds have important functions in planta, possibly functioning as antioxidants, allelopathic chemicals, or in plant-microbe interactions. Determining the biological activities of AHCs should represent a major goal of future research on these compounds.

As it stands now, we have only identified the very first players of AHC biosynthesis. Many fundamental biochemical questions on AHC biosynthesis remain to be answered. For example, how are species or organ-specific compositions of AHCs obtained? Is this related to differential metabolon formation or to catalytic efficiencies of different enzymes? These questions are not only pertinent to AHCs, they are pervasive themes in biochemistry pertinent to the regulation of any biochemical pathway. Having only recently discovered the first transcription factors that regulate suberin, and apparently AHCs, we can now begin to address questions on the transcriptional regulation of AHC biosynthesis. The presence of AHCs in the model plant *Arabidopsis thaliana*, and in plants of agronomic importance (i.e., potato, tomato, etc.), and their putative roles in plant defense present an excellent opportunity to understand the biosynthesis and function of AHCs at a fundamental level, with models like Arabidopsis, but also with applied long-term objectives for agriculture.
